# Phylogeography of post-Pleistocene population expansion in *Dasyscyphella longistipitata* (Leotiomycetes, Helotiales), an endemic fungal symbiont of *Fagus crenata* in Japan

**DOI:** 10.3897/mycokeys.65.48409

**Published:** 2020-03-10

**Authors:** Jaime Gasca-Pineda, Patricia Velez, Tsuyoshi Hosoya

**Affiliations:** 1 UBIPRO, Facultad de Estudios Superiores Iztacala, Universidad Nacional Autónoma de México, 54090, Estado de México, Mexico Universidad Nacional Autónoma de México Estado de México Mexico; 2 Departamento de Botánica, Instituto de Biología, Universidad Nacional Autónoma de México, Mexico City, 04510, Mexico Universidad Nacional Autónoma de México Mexico City Mexico; 3 National Museum of Nature and Science, 4-1-1 Amakubo, Tsukuba, Ibaraki 305-0005 Japan National Museum of Nature and Science Tsukuba Japan

**Keywords:** Divergence, gene flow, geographic information systems, haplotype network, host distribution, intraspecific variation, species distribution modeling

## Abstract

During the Last Glacial Maximum (LGM), drastic environmental changes modified the topology of the Japanese Archipelago, impacting species distributions. An example is *Fagus crenata*, which has a present continuous distribution throughout Japan. However, by the end of the LGM it was restricted to southern refugia. Similarly, *Dasyscyphella longistipitata* (Leotiomycetes, Helotiales, Lachnaceae) occurs strictly on cupules of *F. crenata*, sharing currently an identical distribution. As the effects of the LGM remain poorly understood for saprobiotic microfungal species, herein we identified past structuring forces that shaped the current genetic diversity within *D. longistipitata* in relation to its host using a phylogeographic approach. We inferred present and past potential distributions through species distribution modeling, identifying environmental suitability areas in mid-southern Japan from which subsequent colonizations occurred. Our findings suggest that current high genetic diversity and lack of genetic structure within *D. longistipitata* are the result of recent multiple re-colonization events after the LGM.

## Introduction

Dramatic cyclical glacial advances and retreats characterize the Pleistocene (Bradley 1999). During this epoch the Japanese archipelago underwent important climate changes accompanied by the alternation of dry and wet conditions. Furthermore, by the end of the Pleistocene, during the Last Glacial Maximum (LGM), the advent of a strong cold dry period led to a significant sea level lowering that exposed the entire Seto Sea area (Tsukada 1983). Subsequently, during the Pleistocene-Holocene transition, a rapid warming trend led to an increment of sea level, transforming what had been a long appendage of the Asian continent into an entirely separated archipelago (Aikens and Akazawa 1996).

Strong evidence suggests that, during the LGM, Japan was extensively covered by coniferous forests of *Pinus*, *Picea*, *Abies*, and *Tsuga*. Small populations of *Fagus*, however, were limited to the Pacific coasts of Kyushu and Shikoku (Tsukada 1985). Pleistocene climate and geospatial changes modified the distribution of biomes in this region (Gotanda and Yasuda 2008), causing major migrations and extinctions (Kawamura 2007), and rearranging the geographic distribution of vegetation assemblages (Tsukada 1982b, [Bibr B84][Bibr B85]).

The Japanese endemic canopy tree *Fagus crenata* has been extensively studied in this sense, representing an acknowledged model. Independent lines of evidence suggest that by the end of the LMG, the populations of *F. crenata* were restricted to environmentally stable southern areas (refugia) along the shoreline in southern areas of Japan, and then expanded northwards via two migration routes through the western and eastern shore of Japan (Tsukada 1982a; Tsukada 1982b; Tomaru et al. 1997, 1998; Koike et al. 1998; Fujii et al. 2002; Okaura and Harada 2002). Presently, *F. crenata* is widely distributed from the northern regions in Oshima Peninsula, Hokkaido to southern areas in the Osumi Peninsula, Kyushu, where it occurs sparsely in mountainous regions.

Although the LGM has long been a focal point for research in a wide range of plant and animal taxa, information on the microbial component remains lacking. Fungal phylogeographic analyses are recently increasing (e.g., to identify glacial refugia, re-colonization routes, and interglacial population expansions; Marske et al. 2009, Geml et al. 2010, Leavitt et al. 2013), yet the numbers are still limited (Lumbsch et al. 2008). Several methodological reasons may explain this, including limited taxon sampling, impediments in identification (because of the existence of close relatives with similar morphologies), difficulties in obtaining DNA material (in the case of unculturable symbionts, e.g., Hongoh 2011) and genetically heterogeneous nuclei (such as in some Basidiomycota members e.g. Booth 2014). So, in this sense, culturable and clearly delimited fungal species with wide distribution ranges represent advantageous phylogeographic study models.

*Dasyscyphella longistipitata* Hosoya (Leotiomycetes, Helotiales, Lachnaceae) overcomes the above-mentioned impediments. This culturable ascomycete is highly specific to *F. crenata*, sharing an identical wide geographical distribution throughout Japan (Ono and Hosoya 2001, Hosoya et al. 2010), and so far only known from Japan. Previous ITS-based phylogeographic analyses revealed that *D. longistipitata* forms a genetic continuum comprising three kindred clusters dominated by a single haplotype (named as H12 in Hosoya et al. 2010), which may suggest a recent population expansion (Bandelt et al. 1995; Ferreri et al. 2011). However, the small population numbers, uneven sampling, and a single genetic marker-based analysis hampered the interpretation of the results, leaving unanswered questions as to whether this species occupied the same refugia as its host during the LGM.

In this study, we reconstructed the historical demography of *D. longistipitata* based on genetic variation data, in addition to information on the geographical distribution and ecological attributes for *D. longistipitata* as well as for its host *F. crenata*. The correspondence between both species in their response to major past climate changes was assessed in order to attain a better understanding of the impacts of glacial cycling on the Japanese biota and neglected fungal taxa.

## Materials and methods

### Fungal collection and isolation

**Figure 1.**
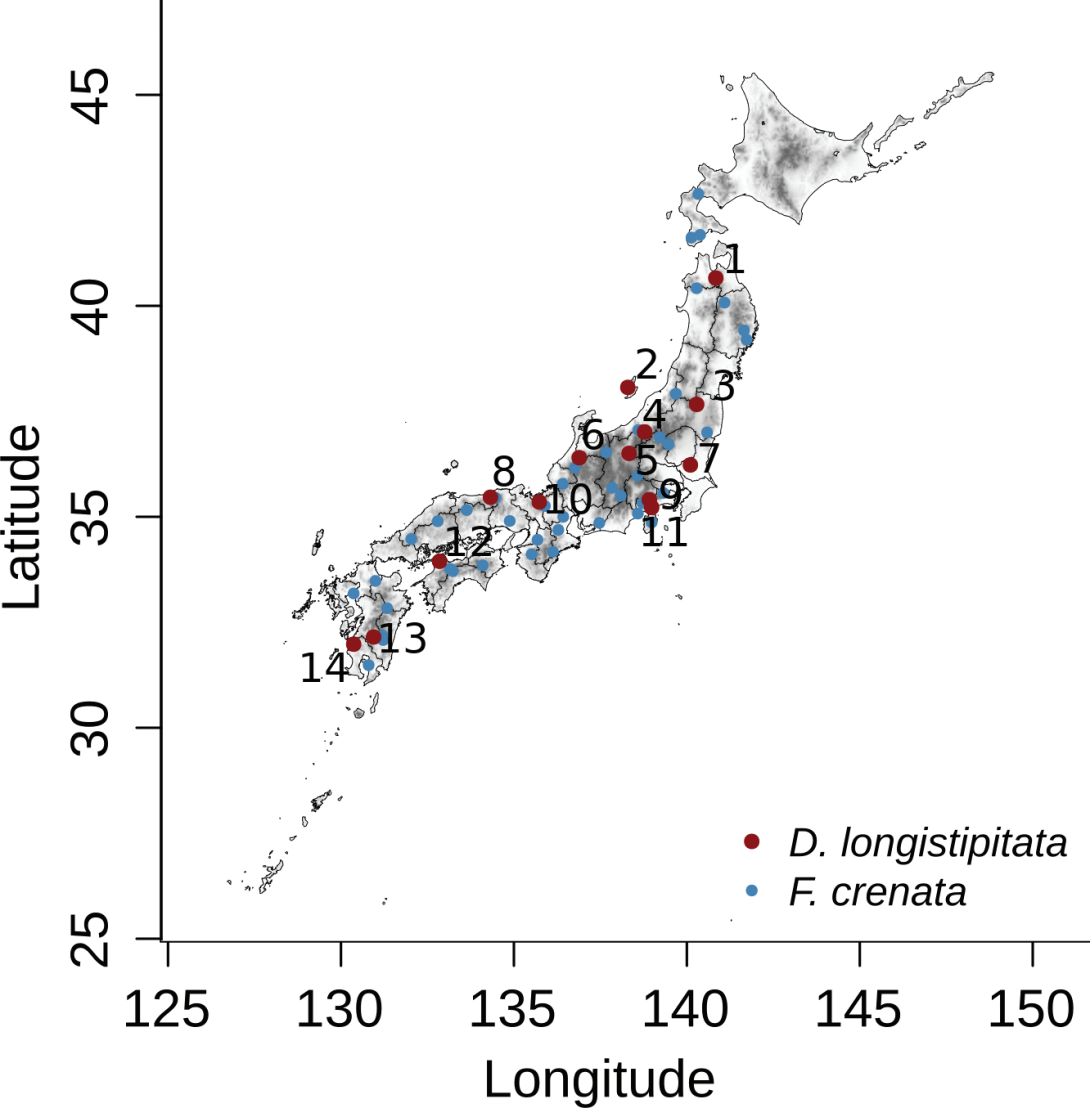
Geographical distribution of the sampling localities for *Dasyscyphella longistipitata* associated with cupules of *Fagus crenata* in Japan. Red dots and numbers correspond to *D. longistipitata*; whereas blue dots represent *F. crenata* study sites from Fujii et al. (2002). For sites nomenclature see Table 1.

A total of 270 fungal collections were sampled during 2010–2018 in 14 localities covering the entire geographical distribution of the host species *F. crenata* from Jogakura (Locality 1) in the northeast to Mt. Shibi (Locality 14) in the southwest (Fig. 1, Table 1). Sampling and isolation procedure followed Hosoya et al. (2010). Briefly, around 20 cupules with *D. longistipitata* apothecia were sampled. Each group of cupules was collectively regarded as a single specimen, and registered under a single number in the fungarium of the National Museum of Nature and Science (TNS) as voucher specimens. Single ascospore isolates were obtained from one of the apothecia occurring on each cupule using a Skerman’s micromanipulator (Skerman 1968), and numbered with a prefix “DL-”.

**Table 1.** Sampling localities, decimal geographic coordinates, and summary statistics for *Dasyscyphella longistipitata* across Japan based on the concatenated ITS and beta-tubulin markers. Significance at p < 0.05 in indicated by *. Latitude and Longitude are indicated in decimal following WGS84 datum; number of isolates analyzed (N), segregating sites (S), haplotype number (h), haplotype diversity (Hd), nucleotidic diversity (Pi), Tajima’s D neutrality test parameter (D).

Number	Locality	Voucher specimen	Collection year	Latitude / Longitude	N	S	h	Hd	Pi	D
1	Jogakura	TNS-F-32166	2010	40.65806, 140.8378	14	56	14	1	0.0106	-1.6175
2	Sado Isl.	TNS-F-38439	2013	38.06667, 138.2928	21	60	21	1	0.0099	-1.3972*
3	Tsuchiyu	TNS-F-46835	2012	37.66302, 140.2851	24	103	23	0.996	0.0090	-1.5928*
4	Mt. Atema	TNS-F-35002	2010	37.01183, 138.7792	17	49	17	1	0.0086	-1.5025
5	Obora	TNS-F-32140	2010	36.50295, 138.3289	16	55	16	1	0.0105	-1.4934
6	Suganuma	TNS-F-32150	2010	36.401, 136.8891	21	70	21	1	0.0101	-1.7464*
7	Mt. Tsukuba	TNS-F-31635	2010	36.22789, 140.1017	20	51	18	0.984	0.0080	-1.5806*
8	Mt. Ougiyama	TNS-F-46830	2012	35.46278, 134.3214	20	63	20	1	0.0098	-1.6550*
9	Mikunitouge	TNS-F-35001	2010	35.40472, 138.9164	20	59	20	1	0.0102	-1.3855
10	Ashiu	TNS-F-46827	2012	35.35045, 135.7326	12	46	12	1	0.0096	-1.5862*
11	Yamakita	TNS-F-32167	2010	35.22131, 138.9831	20	50	20	1	0.0092	-1.1969
12	Mt. Takanawa	TNS-F-32168	2010	33.94556, 132.85	23	77	23	1	0.0111	-1.7589*
13	Mt. Shiragadake	TNS-F-54834	2018	32.15147, 130.9429	21	74	21	1	0.0097	-2.4506*
14	Mt. Shibi	TNS-F-54822	2018	31.98015, 130.3676	21	59	21	1	0.0093	-1.7216
Overall					270	270	255	0.999	0.0096	-2.4491*

### DNA extraction and sequencing

Culturing, DNA extraction and sequencing methods were conducted following Hosoya et al. (2010). Isolates were genotyped by the ITS1-5.8S-ITS2 region using the primer set ITS1 and ITS4 (White et al. 1990), as well as by the beta-tubulin gene using primer set Bt1a and Bt1b (Glass and Donaldson 1995). For the ITS1-5.8S-ITS2 region, PCR procedures were as reported in Hosoya et al. (2010). Whereas, beta-tubulin gene amplification was performed using the following protocol: initial denaturation for 2 min at 94 °C, 35 cycles of 94 °C for 30 s, 51 °C for 30 s and 72 °C for 30 s, and a final extension at 72 °C for 7 min. Total DNA samples were deposited in the Molecular Biodiversity Research Center in the National Museum of Nature and Science, and are available for research upon request. Only material leading to the successful sequencing of both ITS-5.8S and beta-tubulin regions was retained for the analysis (Suppl. material 4: Table S3).

### Genetic diversity and structure

The following population genetics summary statistics were calculated to estimate the amount of genetic diversity: Number of polymorphic (segregating) sites (S), number of haplotypes (h), haplotype diversity (Hd), and nucleotide diversity (Pi). The Tajima’s D neutrality test (Tajima 1989) was computed to infer possible demographic changes (population bottleneck or demographic growth). All statistics were performed using DNAsp v6.12.03 (Rozas et al. 2017). The ITS haplotype numbering followed criteria established by Hosoya et al. (2010), using the prefix “H” and previously reported haplotype numbers to maintain consistency with the previous results. Novel haplotypes were subsequently numbered. For beta-tubulin, prefix “B” was used to designate the sequences. All the haplotype sequences from representative isolates were registered to GenBank as indicated in Suppl. material 5: Table S4.

In addition, to compare our results with further fungal population-level genetic variation estimates, we analyzed ITS and beta-tubulin sequences from NCBI GenBank (by Douhan et al. 2008; Roe et al. 2011; Mo et al. 2018; Cho et al. 2019) for the widely-distributed Ascomycota species: *Colletotrichum scovillei* Damm, P.F. Cannon and Crous, *Aspergillus fumigatus* Fresen., *Ceratocystis montium* (Rumbold) J. Hunt, and *Claviceps purpurea* (Fr.) Tul. We concatenated ITS and beta-tubulin sequences, and calculated nucleotide diversity (Pi) using DNAsp v6.12.03 (Rozas et al. 2017).

To estimate the genetic structure among the 14 sampling localities of *D. longistipitata*, we calculated the AMOVA-based pairwise PhiST (Excoffier et al. 1992), using the R-package haplotypes v1.1 (R Core Team 2018; Aktas 2019). Ten thousand permutations were performed to assess the PhiST statistical significance. The proportion of variance explained by the genetic differences among localities was calculated using the R-package poppr v2.8.3 (Kamvar et al. 2014). To evaluate differences in the genetic variation among localities, we computed a Principal Component Analysis (PCA) at the individual level (isolates), and a Principal Correspondence Analysis (PCoA) using the localities as a grouping factor as implemented in the R-package adegenet v2.1.1 (Jombart 2008). To explore the influence of geographic distance on the distribution of genetic variation, we performed isolation by distance analysis (IBD) using a Mantel test of an Edwards’ distance-matrix against a geographic-distance matrix using R. Furthermore, a haplotype network based on statistical parsimony (Templeton et al. 1992, Clement et al. 2000) was constructed to explore the genetic relationships among haplotypes, using the R-package haplotypes v1.1 (Aktas 2019) with a cut-off probability threshold of 0.95.

### Demographic inference and dispersion routes in *D. longistipitata* and *F. crenata*

We evaluated effective population size changes over time using the Bayesian Gaussian Markov Random Field (GMRF) Skyride Plots (Minin et al. 2008) implemented in BEAST v1.10.4 (Suchard et al. 2018). The best fitting model of sequence evolution was chosen by the Akaike Information Criterion (AIC) using jModelTest2 (Darriba et al. 2012). For this, the ITS and beta-tubulin markers of *D. longistipitata* were concatenated. In addition, we obtained the complete Matk gene sequences reported by Fujii et al. (2002) under the GenBank accession numbers AB046492–AB046523. We used the uncorrelated relaxed molecular clock model to allow rates to vary along branches, using the General Time Reversible (GTR) substitution model with invariable-site proportion and gamma distribution for *D. longistipitata*; and Hasegawa-Kishino-Yano (HKY)+I+G for *F. crenata*. Both analyses run for 500 million generations, and the MCMC convergence was checked using tracer v1.7.1 (Rambaut et al. 2018) discarding the 10% as burning step. Due to the lack of reliable substitution rates (or molecular calibration dates) for fungal ITS and beta-tubulin sequences, we employed the scaled units of effective size and time in the Skyride Plots (Minin et al. 2008). All runs were computed using the CIPRES Science Gateway server (http://www.phylo.org/; Miller et al. 2010).

### Species distribution modeling and spatial phylogenetic analyses

We implemented the analysis of species distribution modeling (SDM) to evaluate the geographical areas of conserved environmental suitability using the BioClim environmental layers (BIO1–BIO19; http://worldclim.org/bioclim; Hijmans et al. 2005) for the present, Mid Holocene, LGM and Last interglacial (LIG; Otto-Bliesner et al. 2008) periods. To avoid possible bias due to highly correlated variables (McCormack et al. 2010; Ortego et al. 2015), we extracted data from the 19 BioClim layers and conducted paired Pearson correlation tests considering a > 0.75 threshold. From each pair of correlated variables, the variable showing more than one significant correlation was discarded. Additionally, we estimated the variance inflation factor using the function “vifcor” of the R- package usdm (Naimi et al. 2014). We obtained 429 geo-referenced records for *D. longistipitata* from the Global Biodiversity Information Facility (http://data.gbif.org; https://doi.org/10.15468/dl.fnvbrv). To reduce bias due to autocorrelation caused by a local overrepresentation of records, we divided the sampling area for each species into cells of 1/10 degrees and selected one point at random from each cell using the R-package raster.

The present and past distribution models were generated using the R package biomod2 v 3.1 (Thuiller et al. 2016) by means of ensemble models (Araújo and New 2007; Qiao et al. 2015). Six standard modeling algorithms were computed: the generalized linear model (GLM; McCullagh and Nelder 1989), generalized boosted model (GBM; Friedman 2001), artificial neural networks (ANNs), random forest (RF; Breiman 2001), multivariate adaptive regression splines (MARS; Friedman 1991), and MAXENT (Phillips et al. 2006). Two independent pseudo-absence sets of 5,000 points were generated at random, and the species records were split into 70% for model training and 30% for model evaluation performance. Using this 70% – 30% criteria, five random replicates were run for all models. A total of 70 independent models (seven algorithms, two background sets and five random replicates) were run to achieve the final ensembles. Model performance was assessed using the area under the receiver operating characteristic curve (AUC; Swets 1988). Final ensembles were built selecting models with AUC > 0.9 and using the committee-averaging criteria.

Areas of conserved environmental suitability across time were delimited as follows: the ensemble layers were transformed to presence/absence considering a threshold of 90% of the distribution of suitability values, and the resulting layers were merged. The area of the resulting layer was classified as highly conserved (overlapping of four layers), medium (at least three overlapping layers), and low (two overlapping layers).

Finally, to estimate the ancestral locations and the spatial dynamics of the two species, we performed a continuous-space phylogeographic analysis using BEAST software. Runs were performed using the molecular clock rates, and substitution models above mentioned. The statistics for spatial traits (i.e., localities) were generated using the Cauchy RRW model. Also, to avoid noise due to duplicate location traits, a jitter window size of 0.01 was used. Posterior to convergence checking, the consensus discrete trait-annotated trees were obtained by means of treeAnnotator using the “Common Ancestor Heights” option and discarding the first 10% trees. Then, this information was used as an input in SPREAD3 v1.0.7 (Bielejec et al. 2011) to geographically visualize the ancestral localities and the possible dispersion routes across the geographic distribution of sampling sites. The KML outputs of the SPREAD3 analysis for *Dasyscyphella longistipitata* and *Fagus crenata* showing colonization trends are available upon request.

## Results

### Haplotype diversity

We analyzed a total of 270 isolates (12–24 per site). Overall, 85 ITS-based haplotypes were identified. The majority was represented by H12 (40.7%), followed by H28 (14.4 %), and H10 (10.7 %), and the 27.8 % were singletons. Both H12 and H28 were found in all the 14 sites, while H10 was found from 12 sites.

In contrast, the beta-tubulin sequences were remarkably diverse, with 224 recognized haplotype patterns. The most frequently observed haplotypes were B4 (3.7 %), B27 (2.2 %), and B75 (1.9 %), and the majority (74.8 %) was occupied by singletons. The ITS and beta-tubulin concatenated sequences ranged from 980–1022 bp, resulting in 255 haplotypes with 270 segregating sites (Table 1, detail for each marker is reported in Suppl. material 2: Table S1). The overall haplotype diversity was 0.999, and the nucleotide diversity ranged from 0.0080–0.0111 among populations. Moreover, overall Tajima’s D parameter was -2.449, with values ranging from -1.3855 to -2.4506 at a population level (Table 1).

### Genetic structure

**Figure 2.**
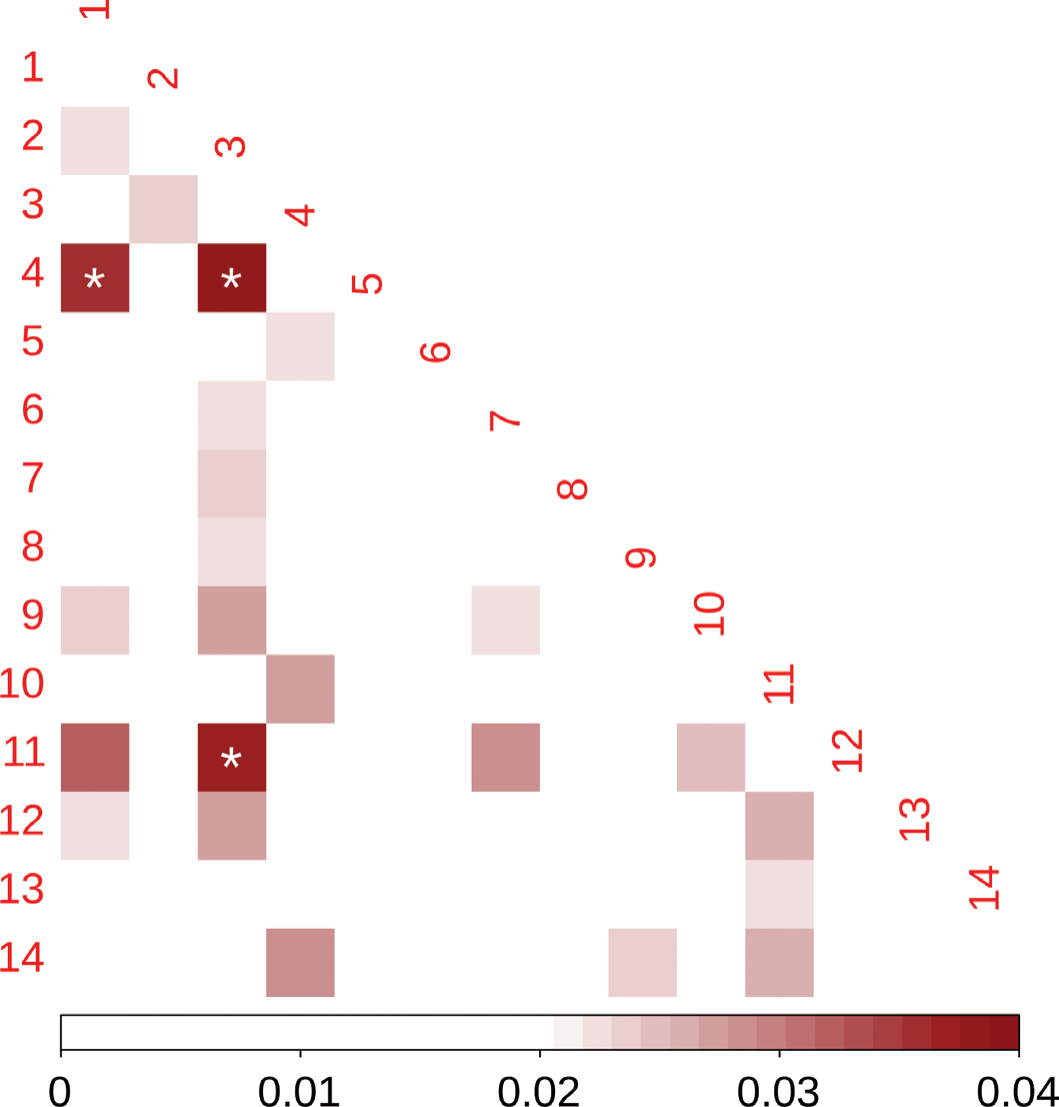
Paired PhiST values for *Dasyscyphella longistipitata* in the 14 studied localities. White asterisks indicate significance at p ≤ 0.05.

**Figure 3.**
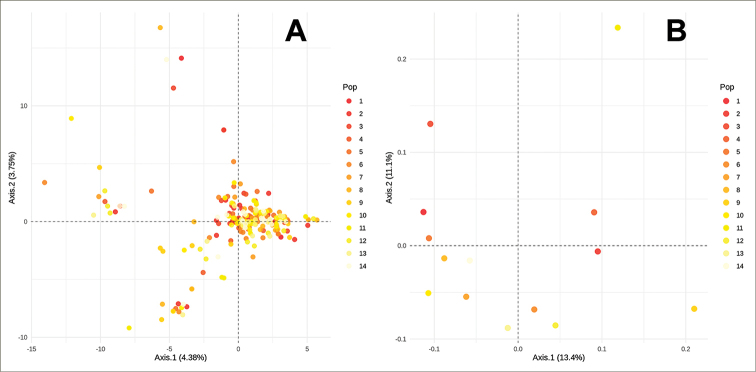
Multivariate analyses of the genetic diversity inferred from ITS and beta-tubulin concatenated sequences of *Dasyscyphella longistipitata***A** Principal Component Analysis (PCA) of genetic diversity at the individual level **B** principal Correspondence Analysis (PCoA) of genetic diversity using the localities as grouping factor. Colors represent the locality of origin arranged as a latitudinal gradient where red represents the further north site.

The paired PhiST yielded low values, ranging from 0 to 0.0358 (Suppl. material 3: Table S2), yet, only three of 96 paired values were significantly different from zero (Fig. 2). Moreover, the AMOVA test showed that 99.99% of the variance was explained by differences between individuals within localities. The individual-level PCA (Fig. 3A) displayed a rambling distribution of individuals without a clear grouping by locality. Although most individuals were stacked around the center of the graph, some individuals with divergent haplotypes were scattered in the four quadrants of the graph; however, they did not belong to neighboring localities. In the case of the locality-level PCoA (Fig. 3B), localities did not show spatial grouping (i.e., neighboring localities were not genetically close). In agreement with the PCoA results, the Mantel test did not show significance (p > 0.5) in the correlation between genetic and geographic distances.

**Figure 4.**
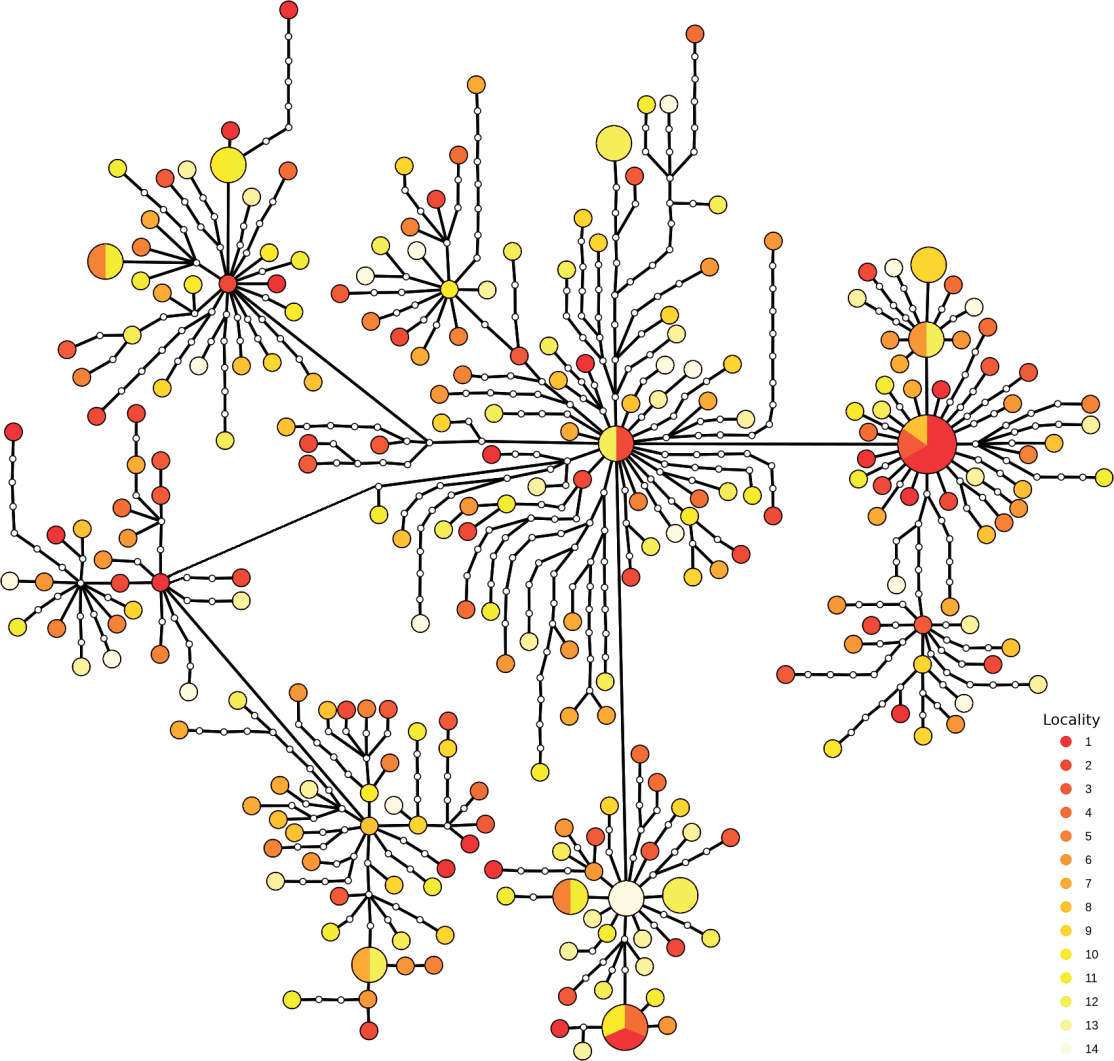
Haplotype network based on the concatenated sequences of ITS and beta-tubulin of *Dasyscyphella longistipitata*. The size of the circles represents the haplotype frequency; white dots represent mutational steps between haplotypes (note that the branches lengths do not correspond to genetic distances). Colors represent the locality of origin arranged as a latitudinal gradient where red represents the further north site.

The haplotype network revealed intricate relationships among *D. longistipitata* haplotypes (Fig. 4), lacking spatial aggregation patterns. Consistently, haplotypes with furthermost geographic distribution were connected by one mutational step. Remarkably, the network displayed multiple star-shaped connections, where central haplotypes did not occur in high frequencies.

### Past demographic inferences

**Figure 5.**
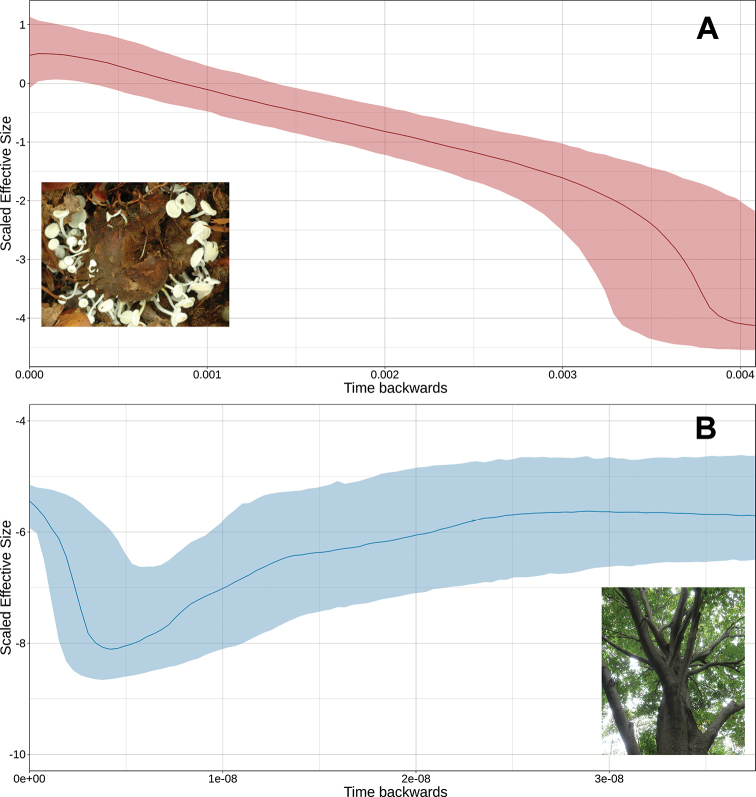
Bayesian Skyride Plot for **A***Dasyscyphella longistipitata* using the concatenated ITS and beta-tubulin, and **B***Fagus crenata*, using the reported sequences in Fujii et al. (2002). The y-axis represents the scaled effective population size (log_10_(Ne*u)), and the x-axis represents time as substitutions per site. Shaded area shows the 95% HPD of the posterior distribution. Solid lines show the median value of effective population size. Dotted shades show the upper and lower 95% highest posterior density. Note that the x-axis in A and B are non-equivalent to each other.

The Skyride plots (Fig. 5) displayed changes in the historical effective size of *D. longistipitata* and *F. crenata*. While *D. longistipitata* showed a continuous and steady growth of the past effective size, the plot of *F. crenata* displayed a three-fashion trend, showing a former stable size followed by a period of constant reduction, and a recent event of population growth. Because the effective size and time scales depend on mutation rates of the different markers, it is important to note that the observed events do not necessarily correspond to the same period in years, nor the actual effective sizes.

### Species distribution modeling

**Figure 6.**
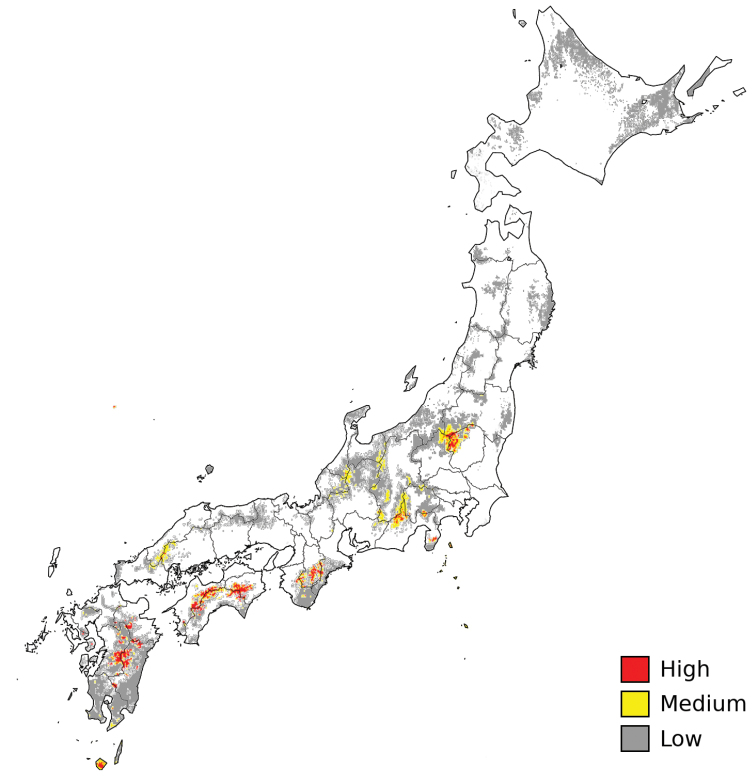
Areas of conserved environmental suitability for *Dasyscyphella longistipitata*, where red is high (overlapping of four layers), yellow medium (at least three overlapping layers), and gray low (two overlapping layers) suitability.

The SDM of *D. longistipitata* resembles the current distribution of *F. crenata* (Nakao et al. 2013). In contrast to the broad area of suitable conditions displayed by present-time projection (Suppl. material 1: Fig. S1A), the past projections revealed areas with non-overlapping geographic areas of environmental suitability (Suppl. material 1: Fig. S1A, B). Moreover, the overlapping geographic areas across periods (current, mid-holocene, LGM and LIG) yielded to limited areas of conserved environmental suitability (Fig. 6), revealing drastic changes in environmental conditions across Japan from the Interglacial period to the present.

### Dispersion trends

**Figure 7.**
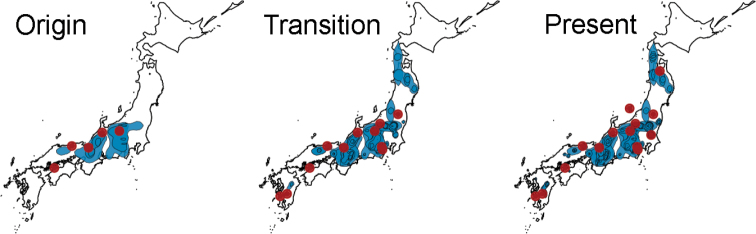
Bayesian continuous-space phylogeographic analyses for *Dasyscyphella longistipitata*, and *Fagus crenata* grouped in three consecutive times. Red dots represent *D. longistipitata* localities, and blue areas are polygons for the nodes of dispersion for *F. crenata*.

Our results on the ancestral localities and dispersion trends, suggested that *D. longistipitata* originated from Mt. Ougiyama (site 8), Obora (site 5), Ashiu (site 10), and Suganuma regions (site 6; Fig. 7, Time 1) in mid-southern Japan. The subsequent events of dispersion took place to the north and south, including the localities of Kyushu (Fig. 7, Time 2). In the specific case of *F. crenata* low genetic diversity (13 haplotypes within 45 sites; Fujii et al. 2002) hampered the colonization tracking at the locality level. However, in good accordance with *D. longistipitata*, the generated dispersion polygons revealed two principal regions for the origin dispersion in central Japan (Fig. 7 Time 1). Following colonization, events were scattered to the north and expanded to the south (Fig. 7 Times 2 and 3). Overall, the SPREAD3 analyses mapped the originating localities for dispersion of both species *F. crenata* and *D. longistipitata* in mid-southern Japan (Fig. 7).

## Discussion

Saprophytic fungi (those obtaining nutrients from dead organic matter) are critical to the dynamics and resilience of ecosystems (Meyer 1994). However, there is tremendous specificity with regard to the types of chemical structure that individual species are capable of degrading (Rodriguez and Redman 1997). To date, the fungal species *D. longistipitata* has been exclusively reported from *F. crenata* cupules, sharing an identical geographical distribution (Hosoya et al. 2010; Ono and Hosoya 2001). Field efforts to obtain material from the co-distributed species *Fagus japonica* as an alternative host (Fang and Lechowicz 2006) have been unsuccessful. In addition to the well-known host specificity in many species of Lachnaceae (e.g., Tochihara and Hosoya 2019), overall this provides further evidence of the strong ecological relationship between *D. longistipitata* and *F. crenata*.

One of the most remarkable results of this study was the unexpected high genetic diversity in *D. longistipitata* (pi = 0.0094; Table 1, Fig. 4). Few population level genetic analyses are available for non-model fungal species, with most of the literature focusing on species of public interest such as phytopathogens (Bennett et al. 2019), human pathogens (Cogliati et al. 2019), insect pathogens (Tsui et al. 2019), and mycorrhiza (Savary et al. 2018). As a result, comparisons are difficult to obtain. Anyhow, the nucleotide diversity estimates for the concatenated ITS and beta-tubulin sequences of *D. longistipitata* were higher in relation to assorted pathogenic Ascomycota including *C. scovillei*, *A. fumigatus*, and *C. montium* (pi = 0.0027, 0.0003, and 0.0041, respectively). Considering that demographic history represents a determinant of genetic diversity, governing effective population size (Ellegren and Galtier 2016), the observed high genetic diversity within *D. longistipitata* may be related to a large effective population size accompanied by a distribution range expansion, resembling what has been reported for its host.

The estimated nucleotide diversity in *D. longistipitata* was lower compared to *C. purpurea* (pi = 0.0101), which is reasonable, as *C. purpurea* comprises three divergent lineages (Douhan et al. 2008). In this context, our results confirmed that *D. longistipitata* represents a well-defined taxonomic entity, useful as a model for phylogeographic studies. This statement is also supported by the ITS-based average genetic divergence (0.99%), agreeing with the canonical 1–3% (−5% Schmidt et al. 2013) threshold for intraspecific variation in fungi (Kõljalg et al. 2013), and specifically 1.96 % SD 3.73 for Ascomycota (Nilsson et al. 2008). Besides, our results did not detect genetic clustering (Fig. 3a), confirming this species forms a single and widely distributed genetic assemblage across Japan (Hosoya et al. 2010).

Several factors influence the genetic structure in fungal populations, due in part to the diverse ecological roles of these osmotrophs. For instance, Prospero et al. (2008) reported high overall F_ST_ = 0.43 in populations of the pathogenic fungus *Armillaria ostoyae* (Romagn.) Herink throughout ~200 km. In this case, biological traits such as host infection ability and reproductive cycle shaped the genetic structure. Furthermore, Barrès et al. (2008) described low but significant genetic structure in *Melampsora populnea* (Pers.) P. Karst. within continental populations in a similar geographic range to the present study. The authors noted positive isolation by distance associated with dispersal constraints, and host preference. In contrast, Velez et al. (2016) described the lack of large-scale geographic structure among populations of the cosmopolitan marine species *Corollospora maritima* Werderm., perhaps as a result of high dispersal potential, or meteorological phenomena like hurricanes.

Even though the sampled localities are distributed across a broad geographic range covering ~1700 km, our genetic structure estimates were practically negligible (Fig. 2, and Suppl. material 3: Table S2). In addition, the lack of correlation between haplotypes relationships and the spatial distribution (Fig. 4), as well as the absence of isolation-by-distance patterns suggest that the lack of structure in *D. longistipitata* is a consequence of the homogeneous distribution of the genetic diversity, instead of a similar genetic composition among localities. Even though dispersion mechanisms remain unknown for *D. longistipitata*, based on its host specificity and field observations, its occurrence as an endophyte is feasible. Under this assumption, a joint seed-vectored dispersion may be possible, opening the opportunity for a broader animal-mediated dispersal (Magyar et al. 2016). This would agree with former reports for the host *F. crenata* (Akashi 1997), and rapid dispersion rates (~100 m/yr) in *Fagus* species under climate change scenario to cope with environmental stress (McLachlan et al. 2005). However, greenhouse experimental and field evidence is required to support this hypothesis.

According to the negative values of the neutrality tests and the star-shaped connections in the haplotype network, the Bayesian Skyride plots revealed that both *D. longistipitata* and *F. crenata* underwent important recent demographic growth events. However, the effective-size of *D. longistipitata* was several orders of magnitude larger than *F. crenata*. For instance, assuming a fixed mutation rate of 1×10^-10 for both species, we would expect a current effective-size of ~36×10^9 for *D. longistipitata*, and 35,000 for *F. crenata*. Moreover, considering a generation time of 10 years for *F. crenata* along with a mutation rate range of 1×10^-11 to 1×10^-12 per site/year (which is feasible for the *matk* gene given the reported mutation rate and the slow evolution rates reported for *Fagaceae* family; Frascaria et al. 1993; Lavin et al. 2005; Barthet and Hilu 2007), the beginning of the demographic growth is estimated for 570 to 5,700 ya; hence stable population size for *F. crenata* would be dated within 2,700 to 27,000 ya. Nevertheless, approximating the time of demographic growth for *D. longistipitata* is problematic.

Aside from the lack of mutation rates for the genetic markers, and precise reports on the number of reproductive events per year (*i.e.*, the generation time), our rough calculations on the estimates for population growth agree with the hypothesis of post-glacial demographic expansion. Furthermore, the demographic increase trend in *D. longistipitata* may be related to a higher mutation rate and shorter generation times. So, growth trends for *D. longistipitata* in the Skyride plots could correspond to a fraction of the time scale in *F. crenata*. In this sense, the mast seeding (large, synchronic seed production) in *F. crenata* (Hiroki and Matsubara 1995), could promote a rapid demographic growth, and high effective size in *D. longistipitata*.

The SDM analyses revealed several restricted areas of climatic stability distributed in mid-southern Japan (Fig. 6). This suggests that *D. longistipitata* and *F. crenata* populations might have had a limited distribution range due to unfavorable past climate conditions in the Pleistocene-Holocene transition, followed by an expansion driven by climatic suitability. According to this hypothesis, the SPREAD3 analysis mapped the origin of dispersion for *D. longistipitata* and *F. crenata* around the predicted areas of climatic suitability in mid-southern Japan (Figs 6, 7), in accordance to former work on chloroplast data of *F. crenata* (Fujii et al. 2002). The statement is also supported by occurrence of the most abundant haplotype (H12B4) in Tsukuba (Site 7), Suganuma (Site 6), and Mt. Atema (Site 4), located in mid Japan among 36.2 to 37.0 N in latitude. However, consecutive dispersion patterns showed slight discrepancies between both species (Fig. 7, Times 2 and 3). This may be attributed to 1) sampling bias or 2) fungal environmental/physiological constraints during ascomata development (e.g., humidity and temperature) as previously suggested (Carré 1964; Fukasawa 2012).

## Conclusions

Modifications in species distribution ranges result from the interaction between ecological and evolutionary processes (Sexton et al. 2009). Our data suggest that drastic past environmental changes marked the genetic diversity within populations of *D. longistipitata*, as exhibited by the magnitude and distribution of the genetic diversity, and historical effective population size fluctuations. Overall, these findings agree with previous reports on the close ecological relationship between the fungus and *F. crenata* (which share an ecological background during Pleistocene-Holocene transition), and the influence of past environmental changes on suitable areas for its distribution.

*Dasyscyphella longistipitata* has been recognized as a fine woody debris decomposer, in particular of cupule litter, an essential component of litterfall in beech forests (Fukasawa et al. 2012). Hence it potentially plays an important role in nutrients cycling and the resilience of the ecosystem. We provide genetic diversity evidence at the population level for this saprophytic fungal species, which is relevant for the conservation and management of forests. Moreover, we confirmed the validity of using host (substrate) data to formulate phylogeographic hypothesis for substrate-specific organisms, which represents an interesting model to explore symbiont relationships under past and future climatic scenarios.

## Supplementary materials

Supplementary material 1 Figure S1. Species distribution modeling (SDM) for *Dasyscyphella longistipitata*. Data type: occurrence Explanation note: **A** Projection for the current major environmental conditions. Color bar represents environmental suitability values, where warmer colors indicate higher suitability, **B** projections for the four time periods analyzed in this study. LGM = Last Glacial Maximum, LIG = Last Interglacial period. The areas represent the upper 90% of the distribution of the suitability values for each period of time. https://binary.pensoft.net/file/388097This dataset is made available under the Open Database License (http://opendatacommons.org/licenses/odbl/1.0/). The Open Database License (ODbL) is a license agreement intended to allow users to freely share, modify, and use this Dataset while maintaining this same freedom for others, provided that the original source and author(s) are credited.Jaime Gasca-Pineda, Patricia Velez, Tsuyoshi Hosoya

Supplementary material 2 Table S1 Data type: statistical data Explanation note: Summary statistics for ITS and beta-tubulin markers, showing the sample size (N), number of polymorphic sites (S), number of haplotypes (h), haplotype diversity (Hd), and nucleotide diversity (Pi). https://binary.pensoft.net/file/388098This dataset is made available under the Open Database License (http://opendatacommons.org/licenses/odbl/1.0/). The Open Database License (ODbL) is a license agreement intended to allow users to freely share, modify, and use this Dataset while maintaining this same freedom for others, provided that the original source and author(s) are credited.Jaime Gasca-Pineda, Patricia Velez, Tsuyoshi Hosoya

Supplementary material 3 Table S2 Data type: statistical data Explanation note: Paired PhiST values between *D. longistipitata* localities (1–14). Bold numbers denote significance at p < 0.05. For nomenclature on sampling localities please refer to Table 1. https://binary.pensoft.net/file/388099This dataset is made available under the Open Database License (http://opendatacommons.org/licenses/odbl/1.0/). The Open Database License (ODbL) is a license agreement intended to allow users to freely share, modify, and use this Dataset while maintaining this same freedom for others, provided that the original source and author(s) are credited.Jaime Gasca-Pineda, Patricia Velez, Tsuyoshi Hosoya

Supplementary material 4 Table S3. List of isolates and their haplotype properties Data type: (measurement/occurrence/multimedia/etc.) Explanation note: All the isolates obtained were numbered with a suffix “DL-”, and their ITS-5.8S and beta-tubulin regions were sequenced. Only isolates that were successful sequenced in both regions were used. In total, 270 isolates were used. https://binary.pensoft.net/file/388100This dataset is made available under the Open Database License (http://opendatacommons.org/licenses/odbl/1.0/). The Open Database License (ODbL) is a license agreement intended to allow users to freely share, modify, and use this Dataset while maintaining this same freedom for others, provided that the original source and author(s) are credited.Jaime Gasca-Pineda, Patricia Velez, Tsuyoshi Hosoya

Supplementary material 5 Table S4. List of accession numbers for ITS-5.8S and beta-tubulin haplotypes Data type: (measurement/occurrence/multimedia/etc.) Explanation note: When a haplotype was obtained from multiple isolate, one isolate was randomly chosen and its sequence was registered. SeqID is for the authors’ private use, and indicated for convenience. https://binary.pensoft.net/file/388101This dataset is made available under the Open Database License (http://opendatacommons.org/licenses/odbl/1.0/). The Open Database License (ODbL) is a license agreement intended to allow users to freely share, modify, and use this Dataset while maintaining this same freedom for others, provided that the original source and author(s) are credited.Jaime Gasca-Pineda, Patricia Velez, Tsuyoshi Hosoya
