# Supplementary figures and images for: Phylogeography of post-Pleistocene population expansion in Dasyscyphella longistipitata (Leotiomycetes, Helotiales), an endemic fungal symbiont of Fagus crenata in Japan
Source: MycoKeys. 2020 Mar 10;65:1–24. doi: 10.3897/mycokeys.65.48409 (PMC7086340; doi:10.3897/mycokeys.65.48409)

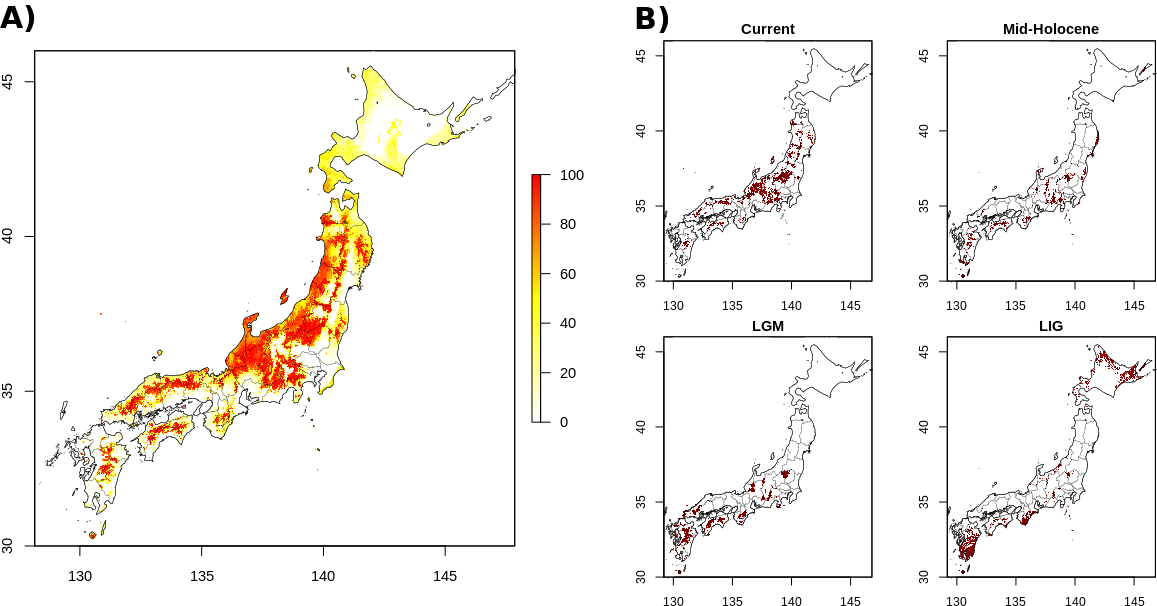

Supplement: Supplementary material 1 [file mycokeys-65-001-s001.tiff]
