# Supplementary material for: Phylogeography of post-Pleistocene population expansion in Dasyscyphella longistipitata (Leotiomycetes, Helotiales), an endemic fungal symbiont of Fagus crenata in Japan
Source: MycoKeys. 2020 Mar 10;65:1–24. doi: 10.3897/mycokeys.65.48409 (PMC7086340; doi:10.3897/mycokeys.65.48409)
Supplement: Supplementary material 2 [file mycokeys-65-001-s002.docx]

Table S1. Summary statistics for ITS and beta-tubulin markers, showing the sample size (N), number of polymorphic sites (S), number of haplotypes (h), haplotype diversity (Hd), and nucleotide diversity (Pi).

| Marker | N | S | h | Hd | Pi | D |
| --- | --- | --- | --- | --- | --- | --- |
| ITS | 270 | 115 | 85 | 0.795 | 0.0029 | -2.81591* |
| B-tubulin | 270 | 154 | 224 | 0.996 | 0.0161 | -2.09799* |
