# Supplementary material for: Phylogeography of post-Pleistocene population expansion in Dasyscyphella longistipitata (Leotiomycetes, Helotiales), an endemic fungal symbiont of Fagus crenata in Japan
Source: MycoKeys. 2020 Mar 10;65:1–24. doi: 10.3897/mycokeys.65.48409 (PMC7086340; doi:10.3897/mycokeys.65.48409)
Supplement: Supplementary material 3 [file mycokeys-65-001-s003.docx]

Table S2. Paired PhiST values between *D. longistipitata* localities (1-14). Bold numbers denote significance at p<0.05. For nomenclature on sampling localities please refer to Table 1.

|  | 1 | 2 | 3 | 4 | 5 | 6 | 7 | 8 | 9 | 10 | 11 | 12 | 13 | 14 |
| --- | --- | --- | --- | --- | --- | --- | --- | --- | --- | --- | --- | --- | --- | --- |
| 1 |  |  |  |  |  |  |  |  |  |  |  |  |  |  |
| 2 | 0.0038 |  |  |  |  |  |  |  |  |  |  |  |  |  |
| 3 | 0 | 0.0073 |  |  |  |  |  |  |  |  |  |  |  |  |
| 4 | **0.0313** | 0 | **0.0358** |  |  |  |  |  |  |  |  |  |  |  |
| 5 | 0 | 0 | 0 | 0.0059 |  |  |  |  |  |  |  |  |  |  |
| 6 | 0 | 0 | 0.0037 | 0 | 0 |  |  |  |  |  |  |  |  |  |
| 7 | 0 | 0 | 0.0075 | 0 | 0 | 0 |  |  |  |  |  |  |  |  |
| 8 | 0 | 0 | 0.0037 | 0 | 0 | 0 | 0 |  |  |  |  |  |  |  |
| 9 | 0.0064 | 0 | 0.0152 | 0 | 0 | 0 | 0.0039 | 0.0006 |  |  |  |  |  |  |
| 10 | 0 | 0 | 0 | 0.0149 | 0 | 0 | 0 | 0 | 0.0004 |  |  |  |  |  |
| 11 | 0.0241 | 0 | **0.0345** | 0 | 0 | 0 | 0.0174 | 0 | 0 | 0.0084 |  |  |  |  |
| 12 | 0.0045 | 0 | 0.0153 | 0.0005 | 0 | 0 | 0 | 0 | 0 | 0.0009. | 0.0130 |  |  |  |
| 13 | 0 | 0 | 0 | 0.0002 | 0 | 0 | 0 | 0 | 0 | 0 | 0.0059 | 0 |  |  |
| 14 | 0 | 0 | 0 | 0.0168 | 0 | 0 | 0 | 0 | 0.0067 | 0 | 0.0132 | 0 | 0 |  |
